# Artificial intelligence applied in identifying left ventricular walls in myocardial perfusion scintigraphy images: Pilot study

**DOI:** 10.1371/journal.pone.0312257

**Published:** 2025-01-17

**Authors:** Solange Amorim Nogueira, Fernanda Ambrogi B. Luz, Thiago Fellipe O. Camargo, Julio Cesar S. Oliveira, Guilherme Carvalho Campos Neto, Felipe Brazao F. Carvalhaes, Marcio Rodrigues C. Reis, Paulo Victor Santos, Giovanna Souza Mendes, Rafael Maffei Loureiro, Daniel Tornieri, Viviane M. Gomes Pacheco, Antonio Paulo Coimbra, Wesley Pacheco Calixto

**Affiliations:** 1 Electrical, Mechanical & Computer Engineering School, Federal University of Goias, Goiania, Brazil; 2 Hospital Israelita Albert Einstein, Sao Paulo, Brazil; 3 Systems and Robotics Institute, Coimbra University, Coimbra, Portugal; 4 Technology Research and Development Center (GCITE), Federal Institute of Goias, Goiania, Goias, Brazil; University of Pisa, ITALY

## Abstract

This paper proposes the use of artificial intelligence techniques, specifically the nnU-Net convolutional neural network, to improve the identification of left ventricular walls in images of myocardial perfusion scintigraphy, with the objective of improving the diagnosis and treatment of coronary artery disease. The methodology included data collection in a clinical environment, followed by data preparation and analysis using the 3D Slicer Platform for manual segmentation, and subsequently, the application of artificial intelligence models for automated segmentation, focusing on the efficiency of identifying the walls of the left ventricular. A total of 83 clinical routine exams were collected, each exam containing 50 slices, which is 4,150 images. The results demonstrate the efficiency of the proposed artificial intelligence model, with a Dice coefficient of 87% and an average Intersection over Union of 0.8, reflecting high agreement with the manual segmentations produced by experts and surpassing traditional interpretation methods. The internal and external validation of the model corroborates its future applicability in real clinical scenarios, offering a new perspective in the analysis of myocardial perfusion scintigraphy images. The integration of artificial intelligence into the process of analyzing myocardial perfusion scintigraphy images represents a significant advancement in diagnostic accuracy, promoting substantial improvements in the interpretation of medical images, and establishing a foundation for future research and clinical applications, such as artifact correction.

## Introduction

Coronary artery disease (CAD) is one of the leading causes of mortality and hospitalization worldwide, including developed countries [1]. In the Brazilian context, CAD was responsible for 43% of deaths attributed to cardiovascular diseases (CVD) and 12% of the total deaths in 2019 [2]. Given that CAD can manifest asymptomatically, prevention and treatment emerge as fundamental strategies for risk stratification and reduction associated with this disease [3]. Considering that early detection and intervention can reduce fatal cases, early diagnosis of CAD is important in medical practice [4]. Various examinations can be used, such as coronary angiography (CA), which is the gold standard for the identification and evaluation of the severity of the condition. However, other non-invasive methods, such as echocardiogram (ECG), computed tomography (CT), cardiac magnetic resonance imaging (CMR), and myocardial perfusion scintigraphy (MPS), offer high sensitivity and specificity for risk stratification [5].

The MPS is useful for visualizing biological functions and cellular perfusion, providing support in the diagnosis and treatment of CAD. This procedure employs the radiopharmaceutical copper (II) tetrafluoroborate tetramibi (99mTc-MIBI) as the primary myocardial perfusion tracer, with images acquired using equipment equipped with cadmium-zinc-telluride detectors (CZT) [6]. Due to the underlying physiological complexity, MPS is prone to clinical limitations resulting from the presence of artifacts. These image irregularities can arise from the patient, the equipment, or technical aspects and can arise at any stage of examination. Therefore, the identification and minimization of these artifacts is important and requires appropriate measures to mitigate their impacts [7, 8].

With technological advancements and personalized patient care, artificial intelligence, particularly deep learning (DL), is revolutionizing cardiology by automating diagnostic interpretation and quantitative analyzes [1, 9]. Liu *et al*. [10] used convolutional neural networks (CNNs) on cardiac images obtained from single-photon emission computed tomography (SPECT) to improve the diagnostic accuracy of CAD. The CNN developed showed higher precision and consistency compared to traditional quantitative methods, although it had lower sensitivity. Integrating CNNs during stress stages enhances the detection of perfusion abnormalities in MPS exams.

Betancur *et al*. [11] introduce a CNN model to improve the automated interpretation of MPS exams. This multicenter study compares CNN results with total perfusion scintigraphy (TPS), the current quantitative method to assess perfusion during the stress and rest phases. Patients underwent stress phases without prior knowledge of the diagnosis of CAD, with coronary angiography performed within 180 days of MPS. The CNN, trained with raw data and polar maps, showed greater sensitivity and specificity compared to TPS in patient-by-patient and vascular obstruction analysis. The results included approximately 62% patients with CAD and 37% obstructed vessels, distributed as 43.7% in the left anterior descending artery, 33% in the circumflex branch and 33% in the right coronary artery.

Liu *et al*. [12] explore the potential of CNNs to improve the detection of perfusion defects in standard-dose medical images. After training and validating CNN, the authors apply the ROC curve to images reconstructed using the Ordered Subset Expectation Maximization (OSEM) technique, attenuation correction (AC), scatter correction, and 3D Gaussian postfiltering, both with and without CNN application, to quantify detection of perfusion defects. The results suggest that the CNN filter applied post-reconstruction can improve the accuracy of defect detection, even in low-contrast defects, compared to Gaussian post-filtering. Furthermore, CNN helps to reduce noise, allowing for reconstruction with fewer post-processing filters, and consequently resulting in higher resolution in the left ventricle.

Olia *et al*. [13] use DL with generative adversarial networks (GAN) to reduce radiopharmaceutical activity in SPECT images for MPS while maintaining image quality and clinical value. This method effectively reduces noise in images with reductions in activity 50% and 25%, although a reduction of 12.5% resulted in low signal-to-noise ratios, which hinders clinical interpretation. In contrast, Hagio *et al*. [14] develop a Deep Learning Attenuation–Corrected (DLAC) algorithm to perform attenuation correction on polar maps, eliminating the need for CT scans. Their results show that DLAC provides comparable attenuation correction to CT, improving diagnostic accuracy compared to non-attenuation-corrected images.

Yang *et al*. [15] use Deep Learning to generate AC-corrected SPECT myocardial scintigraphy images (SPECT*DL*) based on CT-corrected SPECT. The precision of SPECT*DL* compared to CT-corrected SPECT is evaluated using voxel size and segmental analysis, showing artifact reduction in the polar maps. However, performance varies due to attenuation and radiopharmaceutical uptake patterns. Although SPECT_*DL*_ reduces artifacts compared to CT-corrected SPECT, more studies are needed for clinical validation. Kikuchi *et al*. [6] use U-Net and U-Net++ architectures for myocardial extraction from transaxial reconstructions with multi-slice input, reducing extracardiac activity effects. The U-Net++ system with multi-slice input demonstrates high accuracy in myocardial extraction, as indicated by the Dice coefficient.

Other studies, such as the one conducted by El-Taraboulsi *et al*. [16], have explored various CNN architectures to enhance cardiac segmentation in medical images, a component necessary for personalized diagnosis and treatment. Although they have identified gaps in current technologies and pointed out the need for future research, they highlight that the U-Net architecture is widely adopted for this purpose, whereas the No-New-U-Net (nnU-Net) model stands out due to its high precision. Zhu *et al*. [17] conduct a study with 93 patients who underwent computed tomography angiography (CTA) to investigate the performance of the nnU-Net model in detecting and segmenting atherosclerotic plaques in the carotid artery. The results demonstrate that the nnU-Net model offers satisfactory performance, with proven validation, highlighting its utility to diagnose carotid artery stenosis (CAS) and segment carotid atherosclerotic plaques through CTA.

Apostolopoulos *et al*. [18] highlight recent advances in the use of deep learning in cardiac SPECT imaging, summarizing 52 studies in five distinct categories: i) disease classification, ii) noise reduction, iii) attenuation correction (AC), iv) SPECT count estimation, and v) reconstruction. The results indicate that deep learning-based methods achieve levels of agreement with experts, surpassing traditional quantitative approaches. In addition, they are effective for the non-invasive diagnosis of cardiovascular disease, significantly improving image quality and standing out in AC.

Several studies have used emerging technologies such as artificial intelligence (AI) to address medical issues, often focusing on the classification of cardiac anomalies or the diagnosis of disease. However, there is a lack of research exploring the potential of AI in identifying left ventricular walls on myocardial perfusion scintigraphy images. This gap in the literature justifies the present study, whose primary objective is to map the process of identifying the left ventricular walls in these images, incorporating AI techniques. The specific objectives include: i) preparing the patient and perform the myocardial perfusion scintigraphy examination, ii) mapping the process aiming at the implementation of AI as a technique to enhance the quality of scintigraphic images, iii) developing and optimizing the parameters of the Artificial Neural Network, and iv) presenting the results obtained with the approval of a specialist evaluation.

This study adopts an innovative approach by using AI to identify the left ventricular wall to facilitate the future identification of artifacts, assisting doctors in evaluating MPS exam images, and mapping the entire examination process, from the moment the patient enters for the exam until the CNN results. Additionally, it employs optimization methods to find the best configuration of the CNN hyperparameters. The relevance of this study lies in the medical field, as it contributes to reducing the total time required for the annotation and segmentation procedure. For the scientific community, this work stands out for the discoveries that may emerge from the obtained results. This research is economically viable as it is based on a computer system developed by the project team. Its future applications have a positive impact on the fields of cardiology and nuclear medicine.

This paper is organized as follows: Initially, the Section Theoretical Background provides the theoretical basis and necessary concepts for understanding the methodological approach. Subsequently, in Section Methodology, the employed method is thoroughly described, illustrating the complete examination process, from its execution to the evaluation performed by the specialist. The Section Results presents the findings resulting from the application of the proposed method, along with a discussion of the results and implications. Finally, the final reflections and contributions of the study are synthesized in the Section Conclusion.

## Theoretical background

In this section, fundamental concepts are presented to understand the process of identifying the left ventricular walls of the heart in myocardial perfusion scintigraphy images. The discussion covers coronary artery disease, which involves narrowing or blocking of the arteries that supply blood to the heart, and myocardial perfusion scintigraphy, an examination that assesses blood flow to the heart. In addition, common artifacts in this type of image are addressed, providing insight into the challenges of this analysis. Furthermore, a brief explanation of the architecture of convolutional neural networks is provided, an element utilized in the image analysis process.

### Coronary artery disease

Coronary Artery Disease (CAD) is a prevalent and potentially fatal cardiovascular disease, characterized by an inflammatory response leading to the formation of atherosclerotic plaques in the coronary arteries [19]. These plaques, composed of fatty deposits, can vary in size and composition, can become calcified, and cause significant narrowing of the coronary vessels. This process leads to stenosis, which reduces blood flow to the cardiac muscle [20]. Insufficient blood supply to the myocardium can cause symptoms such as transient chest pain and angina, especially during periods of increased oxygen demand [19]. In addition, manifestations such as substernal discomfort, feelings of heaviness and pressure, often radiating to the jaw, shoulders, back, or arms, are common [21].

Angina, one of the characteristic symptoms of CAD, generally disappears when oxygen demand decreases and can be relieved by rest or the use of vasodilator medications [21]. However, in more severe cases, angina can persist even at rest, indicating critical coronary artery obstruction, usually above 90% [19]. CAD can present in acute or chronic forms, including unstable angina and acute myocardial infarction (AMI) in acute form and stable angina in chronic form. In advanced cases, CAD can lead to complications such as heart failure and even death [20]. In some patients, CAD can manifest suddenly and fatally, underscoring the importance of early identification of risk factors to reduce the incidence of unexpected deaths [22].

Cardiovascular disease (CVD) is the result of the complex interplay between genetic and environmental factors, with smoking being the main driver [19]. Understanding these risk factors, adopting a healthy lifestyle, implementing preventive strategies, and performing early diagnoses are necessary to control and eventually eradicate CVDs. In addition, the implementation of preventive measures and specific treatments significantly improves the prognosis of patients with CAD [21]. Although some drugs act directly on the etiological mechanisms to prevent disease progression, more invasive approaches aim to restore blood flow in cases of severe obstructions [20]. Although coronary angiography with X-ray (catheterization) is considered the gold standard for the diagnosis of CAD, other imaging methods such as echocardiography, cardiac magnetic resonance imaging, and myocardial perfusion scintigraphy stand out as promising tools in the investigation and treatment of this condition [22].

### Myocardial perfusion scintigraphy

Myocardial perfusion scintigraphy (MPS) is a widely used imaging examination for the diagnosis, stratification of risk, and prognosis of CAD. It is a tool for the detection of coronary artery stenoses, including the identification of myocardial ischemia and infarctions, through qualitative and semi-quantitative evaluation of regional perfusion patterns [23, 24]. The indications for MPS are determined by the clinical context and the suspicion of perfusion defects or the need to evaluate the response after intervention. This includes situations such as the detection of CAD after evidence, such as: i) elevated troponin, ii) syncope with intermediate risk of CAD, iii) new or worsening angina in diagnosed patients, iv) evaluation of ischemia risk after revascularization, and v) determination of myocardial viability in candidates for revascularization [25].

To undergo the examination, patients are required to wear comfortable clothing and remove any metallic objects that may interfere with the image. In addition, medications and foods containing substances such as methylxanthines and caffeine should be suspended within 24 hours before the procedure [26]. Although there are several options for radiopharmaceuticals available, those using 99mTc technology are preferred due to lower radiation exposure compared to thallium-201 (201Tl), as well as producing superior quality images and lower cost. The cationic compound tetrafluorborate tetramibi cuprous, commercially sold as MIBI or sestamibi, when labeled with 99mTc, accumulates in viable myocardial tissues [24, 27]. Following intravenous administration of technetium-labeled sestamibi, the radiopharmaceutical is withdrawn from the bloodstream and, through passive diffusion, retained within the cells. This uptake of the tracer mirrors the perfusion of the left ventricle, consistent with blood flow in viable tissue [28].

Thus, variations in blood distribution are observed during the resting phases, which represent the baseline condition, and during stress, when the cardiac muscle requires increased oxygenation. This indicates a compromised perfusion in the case of vessel obstruction [5]. In addition to the myocardium, the radioactive material accumulates in other organs and tissues. Physiological biodistribution includes salivary glands, thyroid, liver, gallbladder, small and large intestine, kidneys, bladder, choroid plexus, and skeletal muscle. The primary route of excretion for technetium-labeled sestamibi is hepatobiliary, followed by minor elimination via urinary excretion [29]. Although there are multiple validated protocols, MPS procedures generally involve two stages to be compared: rest and stress. Following one-day protocols, the activity administered for the rest stage varies between 2.5 *MBq*/*kg* and 3.5 *MBq*/*kg*, while for the stress stage, it ranges from 7.5 *MBq*/*kg* to 10 *MBq*/*kg*, representing approximately three times the difference [5].

**Fig 1:**
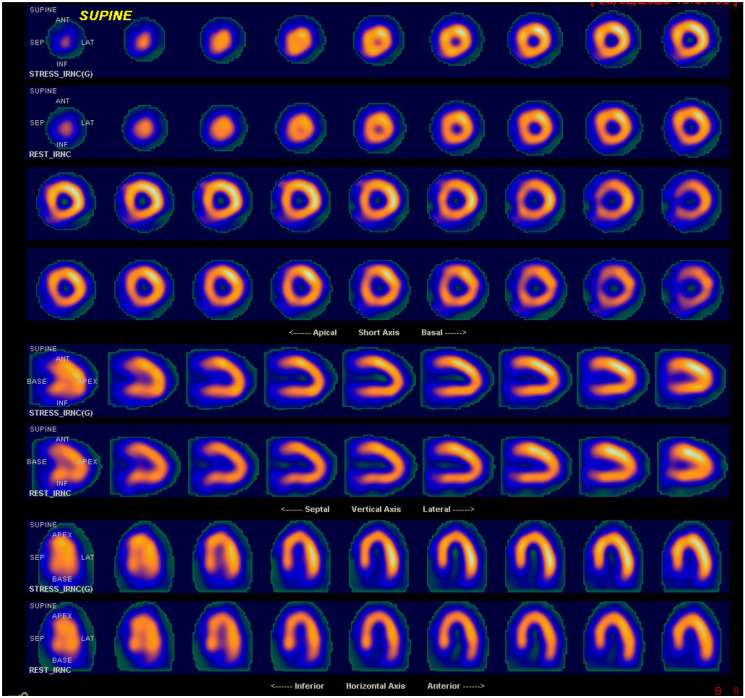
Myocardial perfusion scintigraphy acquired with SPECT.

Acquisitions using the single photon emission tomography (SPECT) technique, as illustrated in Fig 1, begin with intravenous injection of 99mTc-MIBI, followed by ingestion of a high-fat diet and/or cold water to stimulate liver excretion and peristalsis [27]. After this phase, the acquisition of rest images begins. The patient is positioned in the supine or prone position, with the arms raised above the head to avoid attenuation caused by the left arm overlapping the cardiac image [24]. Subsequently, depending on the patient’s condition, the stress stage is initiated [25].

A new tracer administration is performed when the patient reaches the maximum heart rate on the treadmill/bicycle or at the sixth minute of infusion of the pharmacological stress-inducing drug [24, 25]. The acquisition of the image corresponding to this stage is performed between 15 and 45 minutes after stress recovery [25]. Typically, the stress stage is synchronized with the electrocardiogram with SPECT to simultaneously assess perfusion and cardiac function [26]. Exercise on a treadmill ergometer is preferred as it provides valuable prognostic information. However, patients unable to perform physical activities or those with ventricular blockage or left bundle branch block undergo increased heart rate through pharmacological induction, commonly with the vasodilator Dipyridamole. However, patients with contraindications to vasodilators undergo a stress stage with dobutamine, since the mechanism of action to increase blood flow is different [5, 30].

The analysis of the images allows us to observe the physiological mechanism in which the distal myocardial arterioles maintain normal blood flow in the individual at rest. However, under stress conditions, with increased oxygen consumption, there is significant vasodilation of the normal vasculature but little in the stenosed distal vessels, which appears in the images as defects in radiotracer uptake [5]. Scans with ischemic myocardium typically appear normal in the resting stage and with reduced tracer uptake under stress, while a persistent defect in both stages indicates an infarction due to present fibrosis [24]. MPS is considered normal when the tracer concentration is homogeneous in both stages [31].

New equipment dedicated to cardiac imaging, featuring CZT detectors, allows for direct conversion of gamma photons into electrical signals and differentiated iterative reconstruction algorithms, resulting in increased sensitivity and high spatial and energy resolution compared to conventional gamma cameras. This reduces examination time, radiation exposure and, consequently, increases patient comfort and safety [32–34]. Despite these advantages, CZT cameras have limitations, such as the absence of motion correction and integrated attenuation correction, a strategy used to reduce or eliminate artifacts [32].

### Artifacts in myocardial perfusion scintigraphy exams

Myocardial perfusion scintigraphy is a highly precise imaging examination technique with prognostic relevance; however, its precision can be affected by the appearance of artifacts [35]. In nuclear medicine, artifacts are defined as visible abnormalities in images that distort physiological processes or make anatomical structures appear pathological. This reduces the quality of the examination, leading to incorrect results and potentially leading the patient to costly and invasive procedures [24, 36]. These irregularities can arise for various reasons, such as mechanical equipment failures, human error in operation, and patient-related factors. Understanding the mechanisms that can trigger these artifacts is important for correcting them [8]. The technical aspect involves equipment components, image processing, and acquisition protocols. Errors related to the internal structure of the gamma camera can result in artifacts and complicate image interpretation. However, these issues can be avoided through rigorous quality controls and maintenance when necessary [37].

The presence of irregularities in the image after processing can be attributed to the application of incorrect filters or errors in reconstruction, which may result in false-positive or false-negative findings. However, when raw data are obtained accurately, reprocessing may be sufficient to eliminate these artifacts [37]. Regarding the patient, despite advances in equipment, anxiety and discomfort during examination remain challenges. Factors such as fatigue, mobility issues due to previous shoulder surgeries, or flexibility limitations can cause movements during the procedure. Artifacts resulting from patient cooperation difficulties during image acquisition can have a significant impact on examination interpretation and, in some cases, may mimic perfusion defects, leading to false positive results [37, 38]. To minimize these effects, it is necessary that the patient is carefully monitored during image acquisition and fully relaxed, oriented, and placed before examination begins [38].

Artifacts related to patient positioning can also occur, such as the banana-shaped artifact described by Fiechter *et al*. [39], which appears in images acquired with dedicated CZT cameras. This artifact, which appears on polar maps, is generally related to the positioning of the heart outside the center of the field of view and is frequent in obese patients, who are more difficult to center. However, in some cases, this effect can be eliminated from the image by repositioning the heart within the field of view of the equipment [33].

Other artifacts, such as those caused by attenuation of the breast and diaphragmatic tissue, have a direct impact on the myocardial wall. Although diaphragmatic attenuation is common in men and is usually observed in the inferior wall, breast attenuation in the anterior, anteroseptal, or lateral wall of the left ventricle (LV) is frequently observed in women, its severity and extent depend on factors such as breast density, size, and position relative to the LV [8, 37]. Despite being the most common artifacts, they can present in two forms: i) as decreased uptake in the ventricular wall or ii) as high uptake that can obscure the possible perfusion defect in the inferior wall. Attenuation correction can be minimized by acquiring computed tomography images or performing the examination in the prone position [8, 37].

Combined quantitative analyzes of images in supine and prone positions, especially in women, have proven to be highly useful, as they significantly increase the normality and specificity rates of the exams without compromising sensitivity in detecting CAD. This is due to less interference from soft tissues in the prone position [35]. However, during the phase of pharmacological stress, due to the tracer excretion pathway and splenic vessel dilation, the high extracardiac activity in abdominal organs such as the stomach, intestines and liver becomes pronounced, which can hinder the complete visualization of the LV. Strategies to mitigate this excessive uptake include encouraging walking to stimulate peristaltic movement, increasing fluid and high-fat food intake, or extending the image acquisition time interval [28].

Other factors that can induce artifacts in MPS exams are related to the preparation, handling, and storage of the radiopharmaceutical. In order to ensure safety during the procedure, national and international regulations determine that the quality of radiopharmaceuticals is ensured [29]. According to the law, the labeling efficiency of 99mTc-MIBI must be higher than 95%, as otherwise an increase in the concentration of free pertecnetate or colloidal forms may occur in organs such as salivary glands, thyroid, stomach, liver and intestines [8]. Given these variables, understanding the possibility of perfusion defects occurring during image acquisition for CAD diagnosis and how to correct them becomes necessary to achieve the accuracy of MPS exams.

### Deployment of a computational system

The deployment of a computational system in a hospital environment, dedicated to the treatment, storage, management, and distribution of medical examinations, represents a necessary process for the modernization and optimization of the management of clinical information. Known as Picture Archiving and Communication System (PACS), this system is the leading technology in healthcare for image manipulation, including radiographs, tomographies, and magnetic resonances, among others [40]. By replacing traditional radiographic films with digital images, PACS provides quick and secure access to this data for healthcare professionals, as well as facilitating communication between different departments and specialties, leading to a significant improvement in diagnostic and treatment efficiency [41]. Medical images are commonly stored in the Digital Imaging and Communications in Medicine (DICOM) format, a global communication standard that encompasses various areas of healthcare, due to the increasing adoption of medical imaging technology [42].

However, the existence of various syntaxes within DICOM can pose challenges in data integration between different system environments. To overcome this incompatibility, the Vendor Neutral Archive (VNA) disaggregates the original PACS data before migrating it to the new repository system, ensuring the correct interpretation of syntax and facilitating data integration [43]. The VNA serves as a bridge between different image formats and the PACS, employing algorithms to query the received data and perform data normalization tasks. In addition, the system adjusts DICOM-related tags to optimize compatibility [44].

In the context of VNA, privacy preservation is necessary as it encompasses a wide range of medical information and images from various sources. To ensure security and access control, it is important for the VNA to adopt measures such as encryption protocols and user authentication, together with strict privacy policies, ensuring that only authorized healthcare professionals have access to patient data [45]. Furthermore, it is essential for the VNA to be fully compliant with data protection regulations, such as the Health Insurance Portability and Accountability Act (HIPAA), and other laws on healthcare privacy in the corresponding jurisdictions [46]. By adopting these measures, the VNA ensures the integrity and confidentiality of patient information.

Additionally, the anonymizer emerges as an important tool for maintaining the privacy and confidentiality of patients during the process of medical data de-identification. This component removes or replaces personally identifiable information, such as names, identification numbers, and dates of birth, with codes or dummy data [47]. This procedure allows medical records to be used for research, training, or statistical analysis without compromising patients’ identities. This strengthens the security and integrity of the VNA, ensuring that sensitive information remains protected while contributing to technological advancement in the field of healthcare care [48].

Another necessary tool for the implementation of a computer system is the data storage service, a technological solution focused on managing and preserving critical information related to patients’ clinical examinations. An example of such a resource is the Amazon Simple Storage Service (Amazon S3), offered by Amazon Web Services (AWS) [49]. This service provides a highly secure and scalable infrastructure for reliable and accessible data storage, regardless of location [50]. In the hospital context, this means that medical images can be stored efficiently and organized, along with correlated data such as patient information and examination dates. In addition, the service incorporates robust security mechanisms and is compliant with data protection regulations, ensuring the confidentiality and integrity of medical information [51].

With the computer system implemented, it becomes possible to harness advanced technologies to build models that support the medical team. One of these technologies is the convolutional neural network (CNN), which is a specialized form of artificial neural network designed to efficiently process data in grid format, such as medical images [52]. CNN excels at identifying patterns in complex data, using convolutional layers that apply filters to detect specific features in different parts of the image [53]. An example is the U-net network, which adopts CNN architecture and is used in medical image segmentation. This architecture is widely recognized in segmentation tasks. The U-net adopts a structure composed of a contraction (encoding) stage followed by an expansion (decoding) stage. The contraction phase includes several convolutional layers that reduce the spatial resolution of the image while increasing the number of feature channels, capturing informative details [54]. In turn, the expansion stage employs deconvolutional layers to recover the image’s resolution, allowing for the reconstruction of segmentation based on the learned features.

However, U-Net may present limitations in some scenarios, such as those where training data are scarce or heterogeneous, or where segmentation classes are unbalanced or noisy. In this scenario, nnU-net, a variation of U-Net, stands out [55]. What sets nnU-net apart are its unique training and architectural strategies. It employs an efficient training method that automatically adapts pre-processing and post-processing strategies, as well as adapting the morphology of the model to improve segmentation precision [55, 56]. Additionally, nnU-net is tailored to handle medical datasets often constrained in size, a common occurrence in medical segmentation tasks. This makes it particularly suitable for applications that require precise segmentations in medical images, contributing to more accurate diagnoses and more efficient treatments [55].

## Methodology

**Fig 2:**
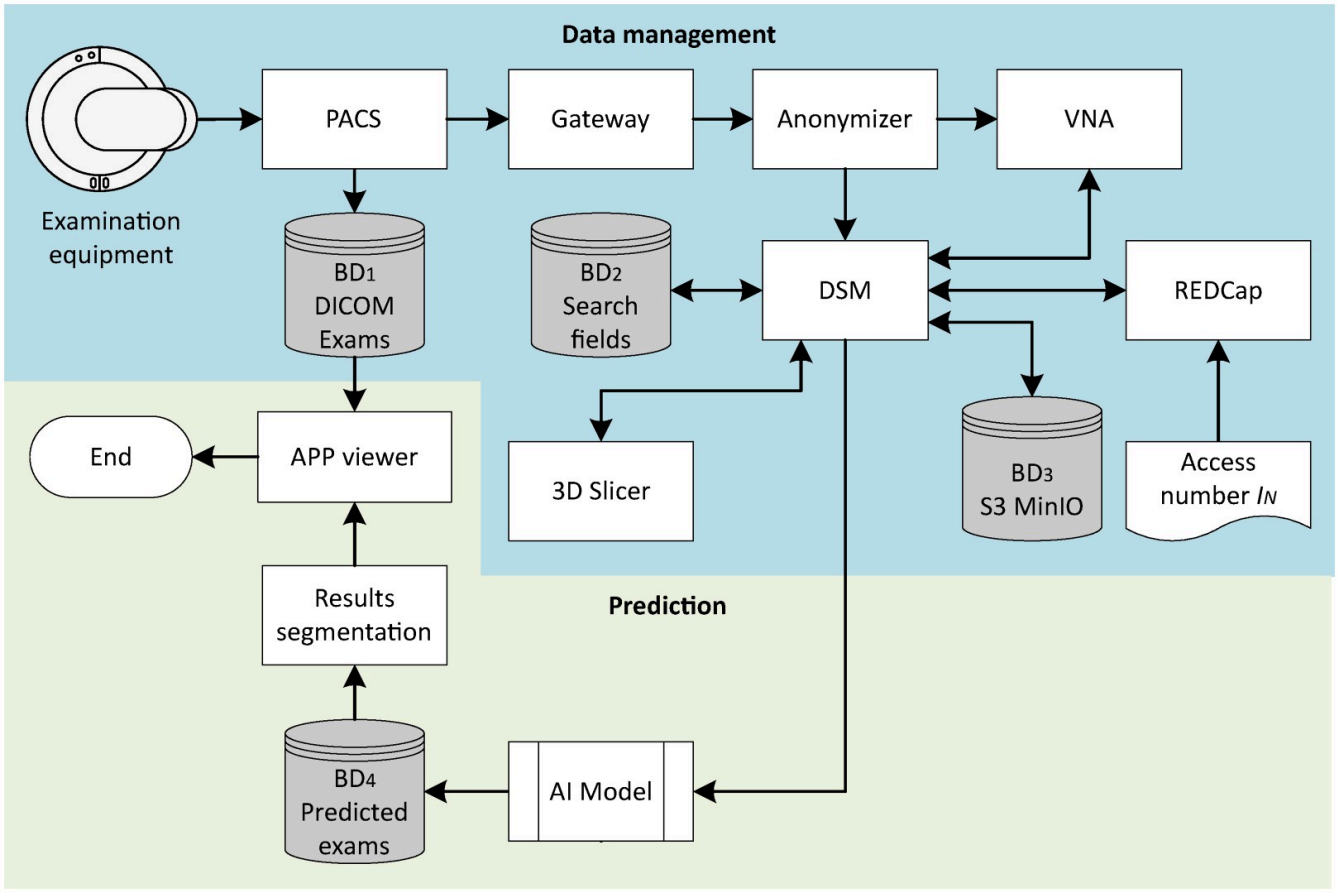
Flowchart of the proposed methodology.

This section describes the methodology developed to map the process of identifying the walls of the left ventricle of the heart in myocardial perfusion scintigraphy (MPS) images using artificial intelligence (AI) analysis. The research has been duly authorized by the ethics committee of the Instituto Israelita de Ensino e Pesquisa Albert Einstein (IIEP), under CAAE number: 65628422.7.0000.0071. The procedure begins with a myocardial examination, in which images are acquired using Discovery NM 530c equipment from General Electric Company Healthcare Ltd. It is a multi-pinhole gamma camera equipped with nineteen Cadmium Zinc Telluride (CZT) semiconductor detectors, organized in a 32 × 32 [*px*] array arranged in an arc so that all pinholes are directed to focus on the heart. The process is concluded when the original scans are compared by specialists with the scans segmented by AI. The complete flow of the proposed methodology is illustrated in Fig 2.

### Protocol for carrying out the exam

This study is prospective in nature and includes all patients referred for the MPS examination, regardless of whether they have a history of coronary artery disease or not. All participants voluntarily signed the Informed Consent Form (ICF), agreeing to provide the examination images after its completion and elaboration of the report. Thus, research is conducted according to the requirements of the ethics committee, and project development begins after anonymization of patient data. In this process, the researchers involved in the analysis do not have access to the patient’s medical history, leaving only essential data for the research conduct.

According to examination guidelines, MPS is performed with the patient in a supine position, following the one-day protocol. The procedure begins with the administration of the 99mTc-MIBI radiopharmaceutical, with the dosage adjusted according to the patient’s weight. After one hour, the rest images are acquired using Discovery NM 530c equipment. The acquisition time is six minutes, with an energy window of 20%, a photopeak of 140 keV, a pixel size of 2.46 × 2.46 mm, a matrix of 32 × 32 pixels, a zoom factor of 1, and subsequently, the images are reconstructed using the Myovation for Alcyone^®^ software from General Electric Company Healthcare Ltd. This software employs the Maximum Likelihood-Expectation Maximization (MLEM) iterative reconstruction algorithm.

### Data management

The images, after being reconstructed by Myovation for Alcyonesoftware ^®^, are digitally sent to the PACS, where a copy of each examination is stored in *DB*_1_ in DICOM format. In each exam, the series of images labeled as recon-rest is selected, representing unblocked images with a number of slices generated from automatic preprocessing of the raw data. Subsequently, only one copy of this series from each exam is transferred to the Gateway, a module that enables the secure transmission of images in DICOM and non-DICOM standards between different equipment, hospitals, and clinics, utilizing Fast Healthcare Interoperability Resources (FHIR) which is the interoperability standard in healthcare designed to facilitate the exchange of information among various systems and applications. This standard was developed by HL7 International, a standardization organization in the healthcare field. The approach is web-based and simplifies the integration of healthcare systems, ensuring uniformity in the exchange of medical data and images [57, 58] and the protocols and standards of interoperability of DICOM.

To ensure the confidentiality of sensitive patient information and control essential data for the formation of the image bank, it is necessary to anonymize the exams. This involves removing any patient identification information. To perform this process, the DICOM Pixel Anonymizer based on the Clinical Trials Processor (CTP) DICOM Pixel Anonymizer (https://github.com/susom/mirc-ctp) is used, a tool designed to handle medical images in DICOM format [59]. The DICOM Pixel Anonymizer utilizes a script file to determine which parts of the image should be removed based on information not contained in the DICOM pixel data. The process involves analyzing the received DICOM objects. The DICOM Pixel Anonymizer checks if the object is an image, and if so, it verifies if the image is uncompressed or has specific compression. If the criteria are met, the anonymizer follows the script to remove the designated parts of the image. Otherwise, the object is passed through unchanged. The regions to be removed are defined by coordinates in rectangular dimensions.

Thus, researchers do not have access to the patient’s identity associated with the exam under analysis. However, at the end of the process, the specialist needs to compare the exam obtained by the equipment with the version segmented by the AI. For this purpose, a form is created using the Web Research Electronic Data Capture (REDCap) platform for data collection and management [60, 61]. This form records the access numbers that identify the anonymized exams, establishing the connection with the exams stored in *DB*_1_. Only the attending physician associated with the patient is permitted to access the identification number *I*_*N*_ in REDCap.

The anonymized exam is forwarded to the VNA, which in this case is the system dedicated to the storage, centralization, and management of medical images. The VNA provides efficient and secure administration of the anonymized data. Both the anonymization process and the VNA direct the data to the dataset Manager (DsM). This facilitates the organization, manipulation, and analysis of data, offering functionalities for data importation, transformation, cleaning, and exploration, optimizing the manipulation process, especially in contexts of large volumes of data. The DsM is connected to both *DB*_2_ and *DB*_3_. The *DB*_2_ is responsible for storing the search fields, which are necessary for conducting the research and remain unchanged during the anonymization process. These fields encompass information such as the gender and age of patients, as well as other relevant data.

The *DB*_3_ corresponds to the S3 MinIO, the Simple Storage Service (Amazon S3), and its function is to store all the data that have been processed in the DsM. The 3D Slicer Platform is then employed for visualization, processing, analysis, and manual segmentation, in which the human operator, usually a healthcare professional or researcher, examines the image and uses specialized software to manually delineate the regions of interest [62]. of medical images. The selection of this platform is supported by the extensive variety of tools available for segmentation of anatomical structures, image registration, generation of 3D models, quantitative measurements, among other functionalities [63, 64]. After the analysis and conclusion of the segmentation, the set of segmented images is transferred to the DsM.

### Prediction model

With the DICOM format image examination available, previously anonymized, manual segmentation of the data is performed, a necessary step for training the AI model. The generation of specialists-produced masks, known as ground truth masks, is important, as the AI model used for automatic mask creation is nnU-Net (https://github.com/MIC-DKFZ/nnUNet) [65]. This technique is based on supervised learning [66]. Manual segmentation is conducted through the 3D Slicer Platform, focusing on the left ventricle. The quality of each mask is verified by different specialists to ensure accuracy. The final step of the segmentation mask construction process is submission to the DsM along with the respective image examinations.

With the image exams and masks available in the DsM, it is possible to train the nnU-Net model. To do so, it is initially necessary to properly name the image files, their corresponding masks, and create JavaScript Object Notation (JSON), a lightweight data exchange format, preferred for transmission between servers and browsers, serving as an alternative to XML [67]. In this work, the function of the JSON file is to describe the dataset so that the processing flow of the nnU-Net can adapt to the problem, detailing important characteristics of the dataset, such as image modality, segmentation classes, and their corresponding labels. The model training process is conducted on an instance provided by Amazon Web Services (AWS), integrated with the Elastic Compute Cloud (EC2) cloud service, belonging to the G4 instance family, optimized for graphics and general-purpose computing workloads. The nnU-Net training is divided into four phases: i) creation of true masks, ii) data preparation, iii) model training, and iv) internal validation.

Regarding the models available for nnU-Net, three options are found: i) U-Net 2D, ii) U-Net 3D Fullres, and iii) U-Net 3D Cascade [65]. In the context of this study, the choice is U-Net 2D, considering the nature of medical data in sliced images, the specific requirements for segmentation, and the search for an optimal balance between resolution, accuracy, and computational efficiency [68]. The internal validation of the model is carried out through the *k*-fold cross-validation methodology [69]. In this approach, the dataset is partitioned into *k* mutually exclusive subsets (folds), and the model is trained and validated *k* times. In each iteration, one of the subsets is designated for validation, while the others are used for training. This procedure ensures that each part of the data is used for both training and validation purposes.

The nnU-Net automatically divides the data into *k* folds, generating *k* distinct models, each trained over multiple epochs. The optimization of nnU-Net hyperparameters is performed using grid search techniques, where different sets of hyperparameters are tested and evaluated in the validation set, with the aim of finding the best combination of hyperparameters that results in optimal model performance using the Dice coefficient evaluation metric *D*_*c*_. This process is repeated iteratively until the optimal combination of hyperparameters is found.

In internal validation, an exclusive subset of data is utilized, different from that used in training, ensuring that the model has not been previously exposed to these data. Performance assessment utilizes *D*_*c*_, which compares the masks generated by the AI model with the ground-truth masks. Following model training and validation, it can be incorporated into the processing pipeline to automatically generate segmentation masks. When the image exam is inserted into *DB*_3_ via the DsM, the AI model is activated to create the corresponding segmentation mask. This mask is stored in *DB*_4_ and becomes accessible in the **viewer APP**. This functionality enables the attending specialist to compare the original exam with the exam containing the mask predicted by the AI model.

### Validation of the proposed system

The validation process of the complete system involves two distinct stages: initially, the quantitative approach where experts perform manual segmentations through the 3D Slicer Platform. Using these segmentations as a reference, we compare with the segmentations generated by the proposed method. This comparison is carried out using the Dice coefficient *D*_*c*_, measuring the quality of segmentation produced by the proposed methodology in relation to the reference segmentation. In the second phase, of qualitative nature, nuclear medicine specialists, experienced in the evaluation of MPS images, examine the images segmented by the proposed methodology. In this context, they conducted the qualitative assessment, providing insights into contour delineation and the efficiency of the model in identifying the walls of the left ventricle.

Experts receive original anonymized exams, manually segmented exams, and simulated AI-generated exams, ensuring that they remain unaware of the identities of those who performed the segmentations (blind). After the qualitative assessment, the experts assign values on a scale of agreement ranging from 20%, 40%, 60%, 80% to 100% for each segmentation. This quantitative assessment complements the qualitative and quantitative analyses using *D*_*c*_, providing an indication of the efficiency of the proposed model. After validation, the proposed model can be employed as a segmentation tool in MPS examinations, eliminating the need for additional validations.

## Results

In this section, we present the results obtained from the application of the proposed methodology, including demographic data, data acquisition for prediction, internal model validation, external system validation, and descriptive statistical analysis, as illustrated in Fig 2. In this pilot study, we used only images from the resting phase obtained without gating (ungated images), following the standardized protocol used at the service where the experiment was conducted. Annotations were made on 50 slices (recon) for each scan, generated using the specific preprocessing of the CZT Discovery 530C camera, which converts pinhole images to SPECT format. This study analyzes raw data in SPECT format, prior to processing with the Myovation for Alcyone software and the QGS/QPS package.

In this study, the terms internal validation and external validation are used as described by Kohavi [70] and Beam & Kohane [71]. Internal validation refers to using a portion of the original dataset, divided into subsets, where the model is trained on one subset and validated on another. External validation involves testing the model with a completely different dataset, not used in either training or internal validation, ensuring the model’s generalizability to new, unseen data. This work does not follow the definitions of Yu, Mohajer & Eng [72], where internal validation involves data collected within the same environment or institution, and external validation refers to the use of data from different sources or institutions outside the environment where the model was developed.

### Obtaining and pre-processing data

The data were collected at Hospital Israelita Albert Einstein, Sao Paulo/Brazil, during February and March 2023. For the MPS procedure, the standard protocol was used with acquisition at rest in the dorsal decubitus position. The inclusion criterion was the signing of the ICF, therefore, all patients who underwent the examination during the collection period and signed the ICF participated in the research. In contrast, the exclusion criterion was the inverse, excluding only patients who did not sign the ICF. Thus, all patients who signed the ICF were evaluated for resting images. All examinations had reports, but for this study only copies of DICOM images were used, the originals stored in the PACS.

Demographic data was collected and recorded in REDCap by the principal investigator, with no other researcher having access to sensitive patient data. After demographic data collection, examinations were anonymized to ensure patient privacy during analysis. Only the principal investigator has access to the *I*_*N*_ associated with the examination, which is in the REDCap form. A total of *N* = 83 patients, ages ranging from 25 to 88 years, the majority being 88% men, as shown in Table 1, where *μ* is the mean, *σ* is the standard deviation, and *Min*, *Max*, **F** and **M** are the minimum, maximum, female and male variables, respectively. Each examination consists of 50 images (or slices) to be segmented, representing a significant challenge in terms of segmentation work.

**Table 1:** Demographic characteristics of participants.

Age [years]			Gender	
*μ* and *σ*	*Min*	*Max*	F	M
62 ± 13	22	88	10–12%	73–88%

**Fig 3:**
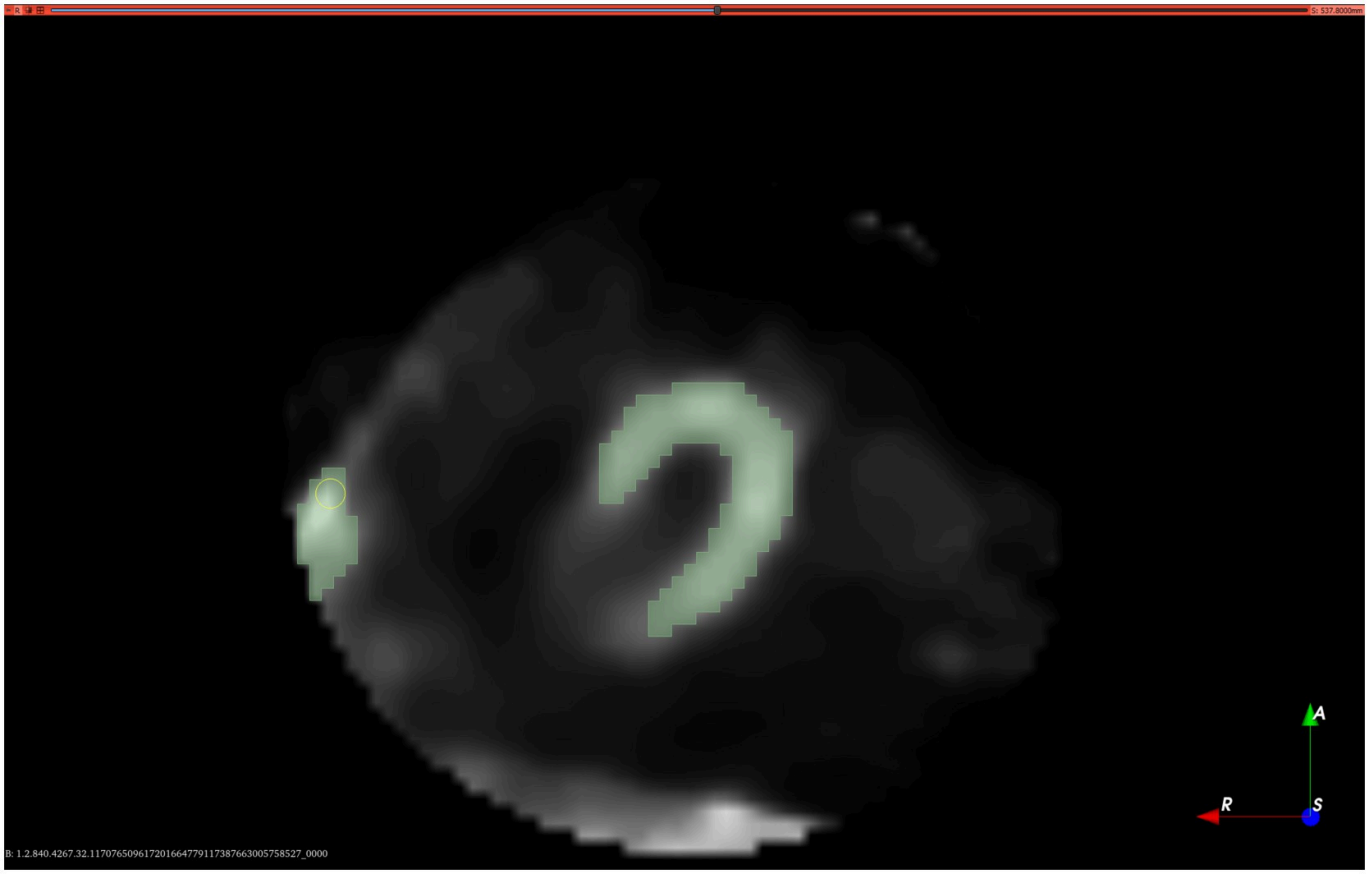
Manual segmentation of the left ventricle produced on the 3D Slicer Platform.

In Table 1, the values of *μ* are accompanied by *σ*, and the absolute values of the number of individuals of each gender are accompanied by the percentage relative to the total number of individuals *N*. All resting MPS images were reviewed by specialists, who identified artifacts in 47 individuals, representing 56.6% of the studied patients. Furthermore, in 37 individuals, which corresponds to 44.6% of *N*, it was necessary to perform additional acquisition in the prone position to ensure image quality for a subsequent medical evaluation, resulting in an increase in the acquisition time of the examinations. After the initial assessment, specialists manually segmented the left ventricular walls in resting images of the patients *n* = 40 using the 3D Slicer Platform. Manual annotations / segmentations were performed by two nuclear medicine biomedical experts with more than five years of experience conducting MPS exams. These professionals segmented the left ventricular walls in all three axes for each of the 50 slices contained in the 83 exams used for the study. These segmentations were considered the gold-standard for comparison. Fig 3 presents an example of manual segmentation of the left ventricle performed in 3D Slicer, with the segmentation mask indicated in green on the vertical long axis (VLA), short axis (SAX), and horizontal long axis (HLA), as well as the representation of the ventricle.

In Fig 3, the Segment Editor module of 3D Slicer was selected, and the Eraser tool was used to erase regions that do not make up the left ventricle, immediately after selecting structures with pixel intensity similar to that of the left ventricle using the threshold tool. The data used were encrypted, and sensitive data were erased in the copies to be used, making it impossible to recover the codes and generated dummy data. Patient data remained only in the original images, and the relationship between the resulting prediction images and the originals occurred only through *I*_*N*_. Regarding data pre-processing, nnU-Net automatically adjusted it, which configured the network and post-processing for image segmentation tasks, eliminating the need for manual adjustments. This capability resulted in high performance and simplified the development of deep learning models. Steps such as normalization according to the image type, resampling for isotropic spacing, cropping, and padding to standardize image sizes, as well as adaptive data enhancement were automatically included.

### Training and internal validation of the prediction model

With the ability of nnU-Net to generate models for each segmentation task provided during training, labels were assigned to each specific type of structure (class), indicating which class the corresponding pixel belongs to. During training, appropriate specific subsets of data were used for validation at each iteration (fold), and thus the number of models generated was determined by the number of segmentation tasks defined in the dataset. In this study, *k* = 5 was established and each model was trained over 1000 epochs. Data were divided into two groups, sequentially separated: images of the first 40 patients were used for training, and the following 43 were used for external validation tests of the prediction system.

**Fig 4:**
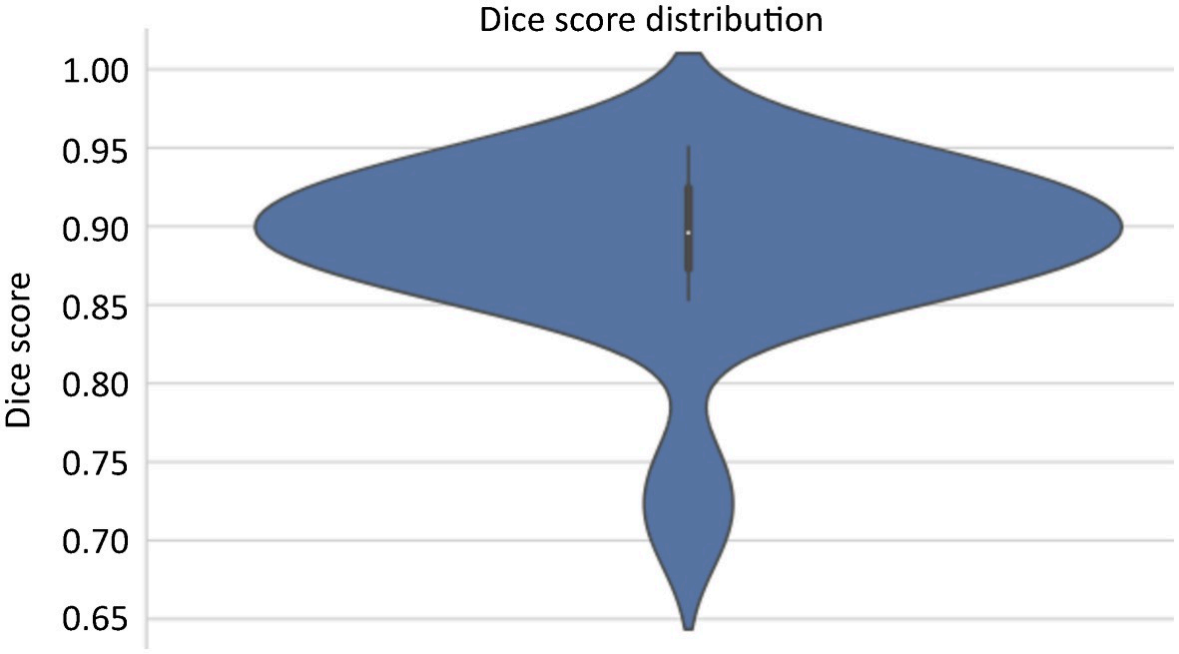
Dice curve with the distribution of scores in the internal validation of the model in the test subset.

In the 2D configuration of nnU-Net, the segmentation model training step was performed using the appropriate subset with 40 images along with their corresponding masks. From this set, 31 images were assigned for training and the remaining nine were reserved for internal validation tests of the model. The *k*-fold cross-validation technique was adopted during training, where each fold resulted in the creation of five unique models. In the test subset, the five generated models were utilized in an ensemble form, combining predictions to capture distinct aspects of the data. A $\bar{D_{c}}=87.01\%$ with *σ* ± 6.10% was achieved. Fig 4 shows the Dice curve with the distribution of overlap measure scores between the predicted segmentation by the model and the segmentation performed in the 3D Slicer over training epochs.

**Fig 5:**
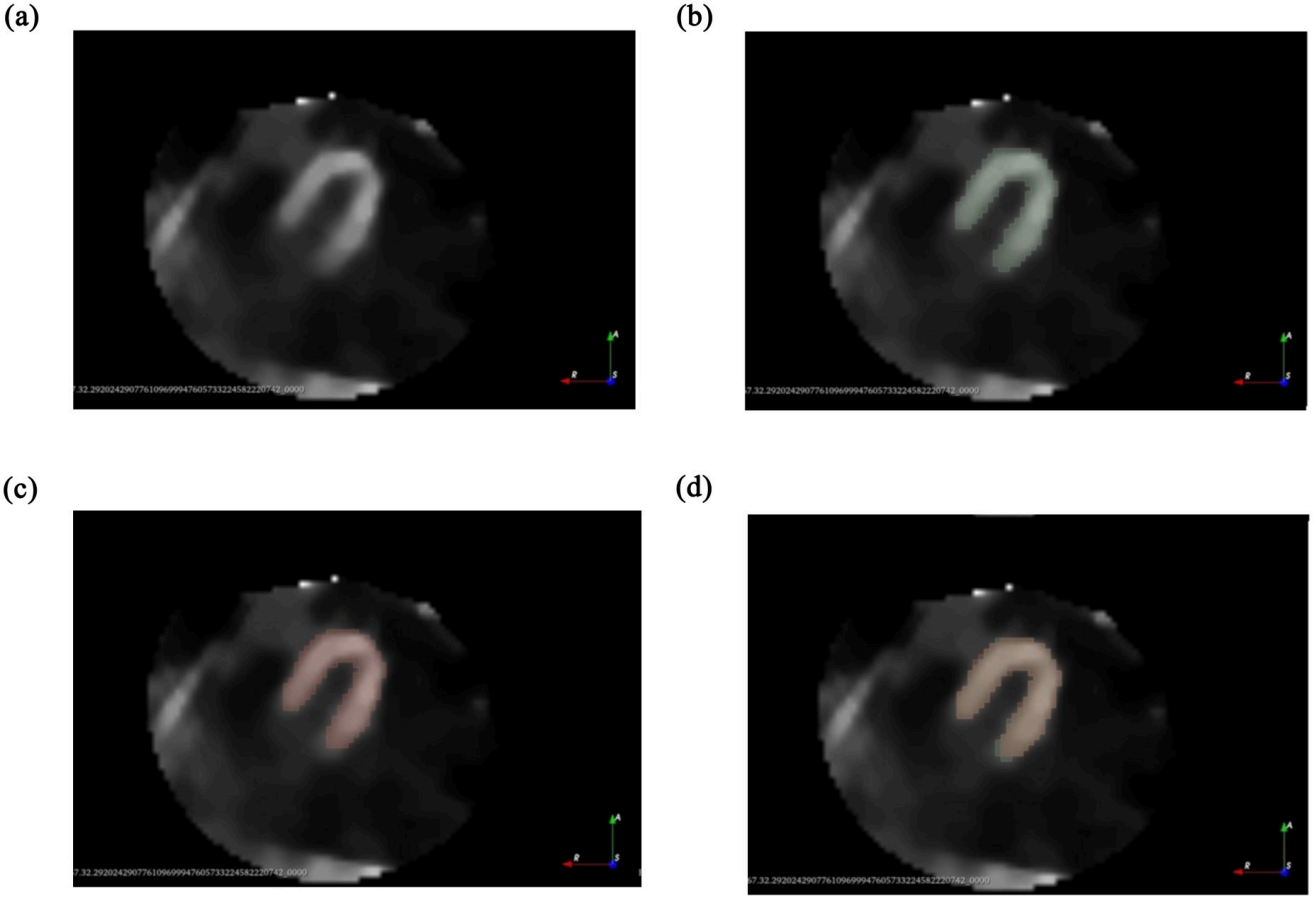
Tomographic section of the left ventricle in the long axis: (a) original, (b) manual segmentation, (c) segmentation with the proposed methodology and (d) overlay of Fig 5(b) and 5(c).

In Fig 5, MPS images were presented as examples to demonstrate the tomographic slice of the left ventricle on the long axis. In Fig 5(a), the original image from the examination was displayed, showcasing the tomographic slice without annotations or masks, showing the characteristic intensity variations of the scintigraphy examination, which are crucial for the starting point in evaluations and segmentations. This original image shows the septal wall, the apex, and the lateral wall of the left ventricle. In Fig 5(b), the manually segmented mask is shown in green, produced in the 3D Slicer platform and used as a reference, highlighting the specific part of the myocardium with perfusion. Finally, in Fig 5(c), the segmentation performed by the proposed method was presented in salmon color, the mask being automatically created by the proposed AI model.

In Fig 5(d), the overlap of the two masks (Fig 5(b) and 5(c)) was presented, which is particularly relevant in this validation context, as it allowed the evaluation of the accuracy of the proposed model compared to manual segmentation techniques. The pixel intensities in the original image were observed to have an impact on the accuracy of manual and automatic masks, and the overlap of the two was used to evaluate the efficiency and discrepancies between the two segmentation techniques. This comparison is important for the internal validation of the proposed model in relation to manual segmentation, considered the gold standard. In this specific example, the similarity between the two images resulted in an average Intersection over Union (IoU) of 0.8.

### External validation of the system

**Fig 6:**
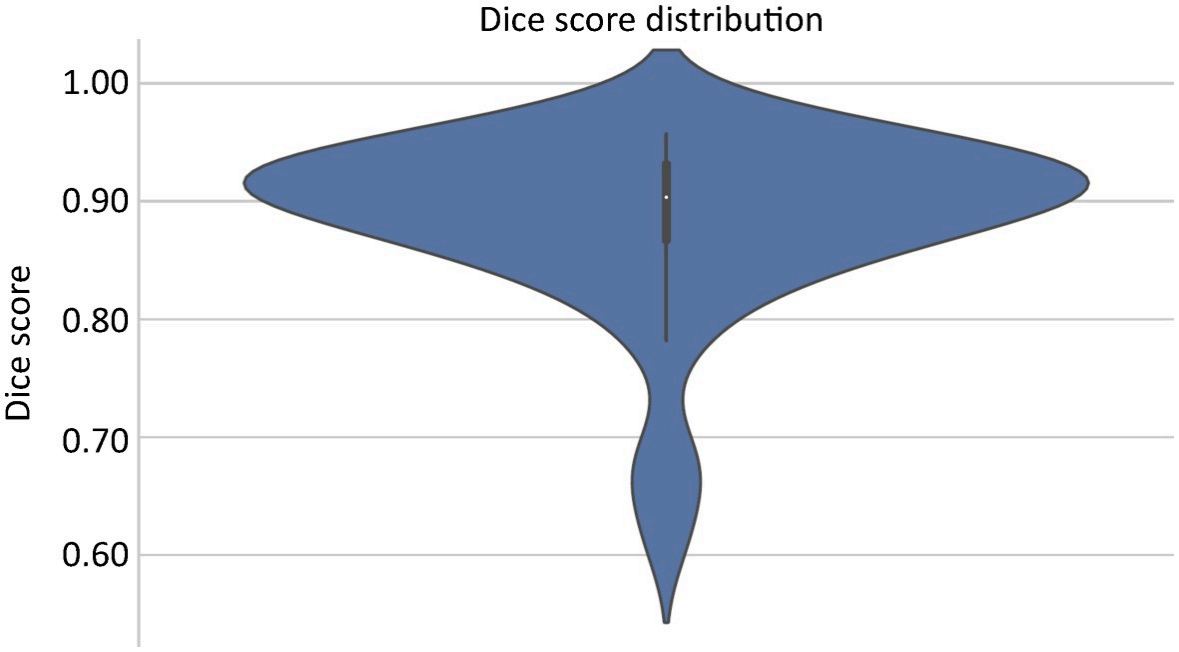
Dice curve with the distribution of scores in the external validation of the system.

External validation is a necessary process to assess the model’s ability to generalize to unseen data, which were not utilized during the training or previous testing phases. This method entails the utilization of a separate dataset, excluded from any training or internal validation phases, with the aim of evaluating the model’s efficiency. The objective is to ensure that the model demonstrates robustness and reliability when applied in real-world contexts, surpassing the limitations of controlled development environments and validating its viability and effectiveness in authentic scenarios. In this pilot project, the *N* examinations were divided into three parts, with 31 used for training, nine for testing (internal validation), and 43 for external validation of the system, as set out in the Table 2. The 43 examinations in the dedicated validation subset were analyzed using the proposed AI tool, achieving an average $\bar{D_{c}}=88.46\%$ with a standard deviation of *σ* ± 7.48%. Fig 6 illustrates the Dice curve with the distribution of overlap scores between the segmentation predicted by the proposed system and the segmentation performed in 3D Slicer by the specialist.

**Table 2:** Number of exams and images for training, testing and external validation.

Dataset	Exams	Images
Training	31	1,550
Testing	9	450
Validation	43	2,150
**Total**	83	4,150

In the subset of 43 examinations, three were randomly selected to be mailed to specialists. Each exam was assigned a designation, such as Test 1, Test 2, and Test 3, organized into separate folders. Each examination consists of 50 slices (volumetric images), requiring each specialist to analyze 150 images. Each folder contained the anonymized volumetric image of the examination, labeled **original**, images of the segmentation mask **manually created** by a specialized biomedical scientist of nuclear medicine, and the images of the mask **generated by the AI tool**, both focused exclusively on the walls of the left ventricle. The masks were randomized and grouped into two sets, called Mask 1 and Mask 2, ensuring that the attending medical specialists *M*_*N*_ responsible for evaluating the proposed tool were unable to distinguish which mask was produced by AI or biomedical specialist.

**Fig 7:**
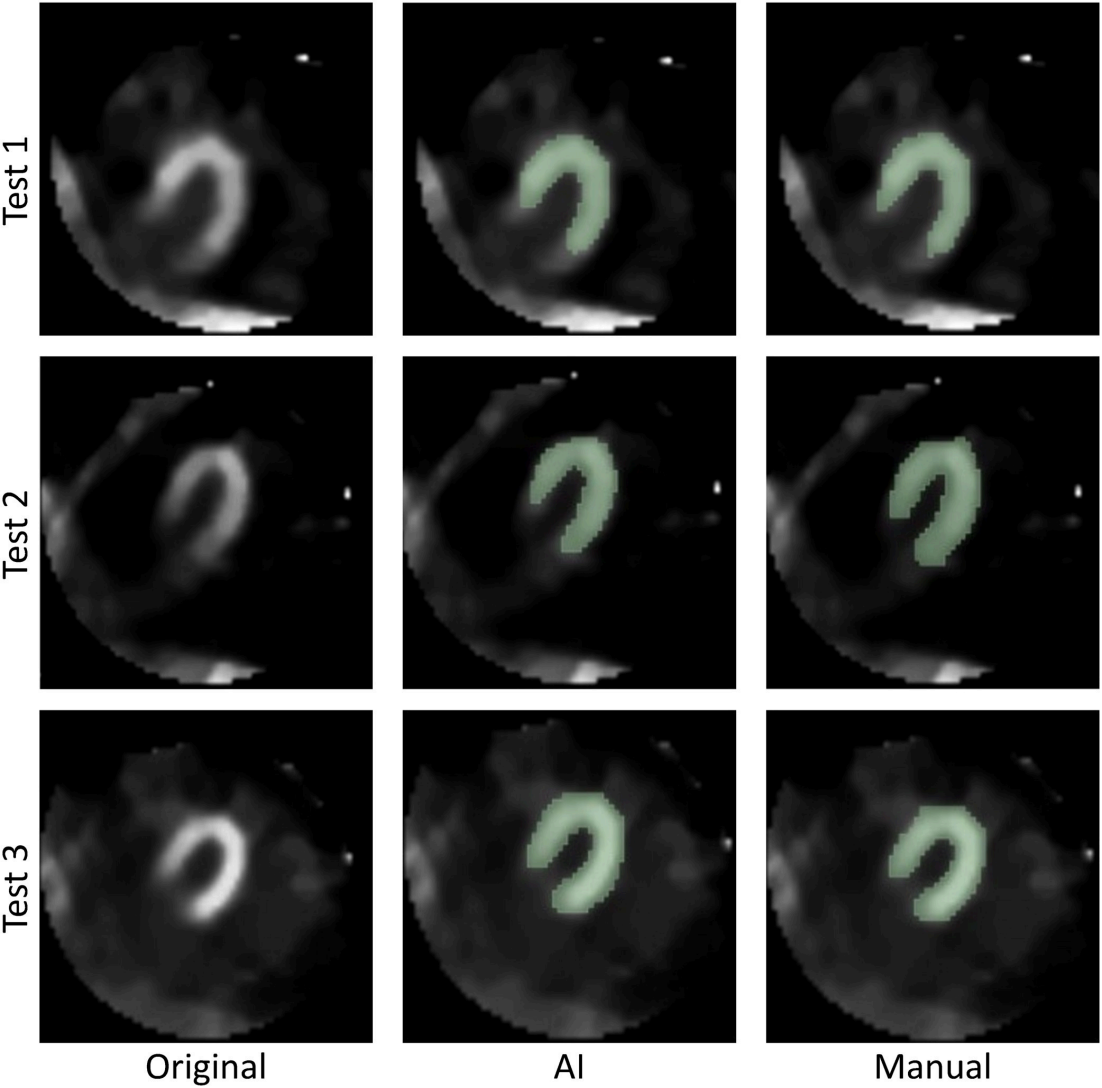
Images chosen at random and forwarded to the *M*_*N*_ in charge of evaluation.

Three nuclear medicine specialists, $M_{N_{1}}$ with 18 years of experience, $M_{N_{2}}$ with 20 years of experience, and $M_{N_{3}}$ with 12 years of experience, independently and blindly evaluated the delineation of the walls of the left ventricle in each mask, following the criteria defined in the methodology. This analysis showed that, most of the time, there was a high degree of agreement among specialists, with evaluations indicating a agreement between 80% and 100% in the delineation of the masks in both groups, as shown in Table 3. Fig 7 visually presents the results, displaying Test 1, Test 2, and Test 3 in the rows, and the original images, the masks generated by AI, and the masks created manually by specialists in the columns, respectively.

**Table 3:** General medical evaluation of the tests.

Scale items	AI	Manual
I agree 100% with the mask	4	4
I agree 80% with the mask	5	4
I agree 60% with the mask	0	1

Table 4 presents the individual assessments of the tests and their agreements. In the qualitative analysis conducted by the medical specialists *M*_*N*_, it was observed that there was at least agreement 80% about the position of the masks generated by the proposed tool. In Test 1, which recorded the greatest divergence among the evaluators, the lowest score was assigned to the manual mask. The discrepancy was mainly due to the proximity of the heart to the artifact zone, resulting in a 60% agreement on the position of the manual mask.

**Table 4:** Individual medical assessment by test.

	Evaluation					
	Test 1		Test 2		Test 3	
Scale items	AI	Manual	AI	Manual	AI	Manual
I agree 100% with the mask	1	0	1	2	2	2
I agree 80% with the mask	2	2	2	1	1	1
I agree 60% with the mask	0	1	0	0	0	0

### Discussion

This study explored the application of Artificial Intelligence (AI), highlighting the use of Convolutional Neural Networks (CNN) and nnU-Net to identify the walls of the left ventricle of the heart in images of myocardial perfusion scintigraphy (MPS). The investigation achieved the desired segmentation precision, as demonstrated by *D*_*c*_ = 87% and an average *IoU* = 0.8. These metrics indicate agreement with manual segmentations performed by expert professionals, highlighting significant advancements in diagnostic interpretation and surpassing some traditional methods, such as manual segmentation.

The synergy of this study with the existing literature underscores the relevance and applicability of AI in medical image analysis. Previous research, implicitly referenced, has suggested the potential of AI to enhance diagnostic accuracy and identify the walls of the left ventricle, enriching the diagnosis of CAD. Works such as those by Liu *et al*. [10], Liu *et al*. [12], Slomka *et al*. [73], and Eisenberg *et al*. [74] have highlighted AI’s potential to improve image interpretation and CAD detection, providing new insights into left ventricle wall identification and segmentation accuracy enhancement. This proposal advances in this direction by indicating the practical efficiency of AI in identifying left ventricle walls, corroborating the feasibility and effectiveness of the AI model in clinical settings, validated both internally and externally.

The implementation of the proposed model has been shown to be pioneering in the automated interpretation of MPS exams, shedding new light on the accuracy of the identification of the left ventricle wall, a significant contribution to the field. Furthermore, the high agreement in the evaluations of medical experts demonstrates the ability of the model to tackle the complexity and variability of medical image interpretation, overcoming challenges such as those observed in Test 1.

Methodological challenges, such as data collection and preprocessing in clinical settings, were overcome despite the difficulties imposed by the need for anonymization and compliance with ethical protocols. The study also acknowledges limitations, such as reduced sensitivity in certain cases, which point to the need for future improvements to the model to address these issues.

This study stands out for its methodological approach, from data collection to MPS image analysis, using advances in AI to improve the diagnosis of CAD. The optimization of CNNs and the adoption of advanced techniques such as U-Net++ to mitigate effects of extracardiac activity mark significant progress in the field. Thus, in addition to enriching diagnostic practice in cardiology and nuclear medicine, this work proposes an economically viable solution for the clinical incorporation of these technologies, emphasizing the importance of technological advancements in the field of healthcare. The next step, after the implementation of the computational system throughout the hospital network, is artifact correction, as once the walls of the left ventricle are accurately identified, the tool promises to identify artifacts.

This is a pilot study conducted to train the neural network in the identification of the left ventricular wall and to map the processes from the obtaining of clinical images to the application of the convolutional neural network. It corresponds to the first phase of a larger project whose ultimate goal is to assist professionals performing MPS in identifying scans where there is doubt regarding the presence of artifacts that may interfere with image quality. In this initial phase, it was necessary to test the artificial intelligence tool specifically to identify the left ventricle wall. For this reason, only part of the collected exams was used. This approach allowed for a focused and controlled validation of the model’s efficiency before expanding the study to a larger database.

The choice of a smaller sample was strategic in order to ensure a controlled scenario. However, this limitation implies that the results obtained so far should be interpreted with caution and subsequent studies with larger samples are necessary to confirm the generalization of the findings. It is acknowledged that the sample size limitation restricts the immediate applicability of the results. However, this pilot study provides a solid foundation for future research and ongoing project development, paving the way for the implementation of a solution in MPS. Another limitation is the use of thresholding, which heavily depends on the selection of an appropriate threshold value, which can be difficult to determine automatically and may lead to inaccurate segmentations. Thresholding does not consider the spatial context of the pixels, which can be challenging in images where the pixel intensities do not clearly distinguish the structures of interest from the background. This will be addressed in the second phase of this project.

## Conclusion

This study addressed the identification of the left ventricular walls of the heart in MPS images using artificial intelligence techniques. Investigating the challenge posed by CAD, one of the leading causes of global mortality, this work aimed to improve the diagnostic accuracy and interpretation of MPS images, crucial for the treatment and prevention of CAD. The main hypothesis that AI could enhance the identification of left ventricular walls in MPS images, surpassing the limitations of operator-dependent analysis, was corroborated. This was observed by the results, such as the Dice coefficient of 87% and the average Intersection over Union (IoU) index of 0.8, indicating agreement with the manual segmentations performed by experts.

The proposed objectives were achieved, with emphasis on the development and optimization of an AI model efficient in identifying the left ventricular walls, as well as the comprehensive mapping of the MPS examination flow. The methodology used, which ranged from patient preparation to final analysis by experts, demonstrated the feasibility and efficiency of incorporating advanced technologies into medical practices. The most significant outcomes of the study were the accuracy in image segmentation and consistent validation of the model both internally and externally, reflecting the success of the investigation. These results not only reaffirm the utility of AI in the fields of radiology and cardiology, but also pave the way for future research focused on further refinements and broader clinical applications.

Thus, it can be concluded that this study represents a significant contribution by applying AI to enhance the identification of left ventricular walls, aiding in the interpretation of MPS images. In addition to overcoming technical and methodological challenges, the research provides important advances for medical practice, notably improving and optimizing processes. In this way, it offers a solid foundation for the continued development of AI techniques in healthcare. Consequently, once the heart walls are accurately identified, the tool demonstrates potential to detect and correct artifacts in MPS images.
